# The Association Between School Closures and Child Mental Health During COVID-19

**DOI:** 10.1001/jamanetworkopen.2021.24092

**Published:** 2021-09-03

**Authors:** Matt Hawrilenko, Emily Kroshus, Pooja Tandon, Dimitri Christakis

**Affiliations:** 1 Department of Psychiatry and Behavioral Sciences, University of Washington School of Medicine, Seattle; 2 Seattle Children’s Research Institute, Center for Child Health, Behavior and Development, Seattle, Washington; 3 Department of Pediatrics, University of Washington, Seattle

## Abstract

**Importance:**

In-person schooling has been disrupted for most school-aged youth during the COVID-19 pandemic, with low-income, Black, and Hispanic populations most likely to receive fully remote instruction. Disruptions to in-person schooling may have negatively and inequitably affected children’s mental health.

**Objective:**

To estimate the association between school closures and child mental health outcomes and how it varies across sociodemographic factors.

**Design, Setting, and Participants:**

This cross-sectional population-based survey study included a nationally representative sample of US adults aged 18 to 64 years with at least 1 child in the household. The survey was administered between December 2 and December 21, 2020, via web and telephone in English and Spanish. Participants were recruited from the NORC AmeriSpeak panel, an address-based panel with known probability sampling and coverage of 97% of US households.

**Exposures:**

Schooling modality (in person, fully remote, or hybrid), household income, age.

**Main Outcomes and Measures:**

Child mental health difficulties were measured with the parent-report version of the Strengths and Difficulties Questionnaire, with small, medium, and large effect sizes defined as 1.3-, 3.3-, and 5.2-point differences, respectively.

**Results:**

A total of 2324 adults completed the survey. Overall, 1671 respondents (71.9%) were women, 244 (10.5%) were Black, 372 (16.0%) were Hispanic, and 421 (18.1%) had a high school education or less. Children attending school in-person had higher household incomes (mean difference, $9719; 95% CI, $4327 to $15 111; *P* < .001) and were more likely to be White compared with those attending remotely (366 of 556 [65.8%] vs 597 of 1340 [44.5%]; *P* < .001). Older children in remote schooling had more mental health difficulties than those attending in-person schooling (standardized effect size, 0.23 [95% CI, 0.07 to 0.39] per year older; *P* = .006), corresponding to small effect sizes in favor of in-person schooling for older children and very small effect sizes favoring remote schooling for younger children. Children from families with higher income benefitted more from attending schools in-person compared with their peers from families with lower income (B = −0.20 [95% CI, −0.10 to −0.30] per $10 000-increase in annual income; *P* < .001), although this advantage was not apparent for children attending hybrid school (B = −0.05 [95% CI, −0.16 to 0.06] per $10 000-increase in annual income; *P* = .34), and directionally lower but not significantly different for children attending remote school (B = −0.12 [95% CI, −0.04 to −0.20] per $10 000-increase in annual income; *P* < .001). Learning pods fully buffered the associations of hybrid schooling (*d* = −0.25; 95% CI, −0.47 to −0.04) but not remote schooling (*d* = 0.04; 95% CI, −0.10 to 0.18) with negative mental health outcomes.

**Conclusions and Relevance:**

The findings of this study suggest that older and Black and Hispanic children as well as those from families with lower income who attend school remotely may experience greater impairment to mental health than their younger, White, and higher-income counterparts. Ensuring that all students have access to additional educational and mental health resources must be an important public health priority, met with appropriate funding and workforce augmentation, during and beyond the COVID-19 pandemic.

## Key Points

**Question:**

Is there an association between school closures and child mental health outcomes, and how does it vary by key sociodemographic characteristics?

**Findings:**

In this survey study of 2324 adults with at least 1 school-aged child, a small association between school closures and worse child mental health outcomes was observed, with older children and children from families with lower income experiencing more mental health problems associated with school closures. Children from families with lower income and those belonging to minority racial/ethnic groups were most likely to experience school closures.

**Meaning:**

These findings suggest older and Black and Hispanic children as well as children from families with lower income who attend school remotely may experience disproportionate mental health difficulties.

## Teaser

This survey study estimates the association between school closures and child mental health outcomes and examines how this association varies across sociodemographic factors.

## Introduction

School closures have been widely used as a risk mitigation practice during the COVID-19 pandemic,^1^ with in-person schooling disrupted for most school-aged youth. In the United States, estimates suggest that in November and December 2020, approximately half of students in kindergarten through 12th grade attended school fully remotely, 19% attended school in a hybrid model that combined remote and in-person instruction, and 28% attended school fully in-person.^2^ There was socioeconomic variability in instructional modality, with low-income, Black, and Hispanic groups the most likely to attend fully remote school.^2^

Pandemic-related disruptions may be negatively, and inequitably, affecting children’s mental health. One-third of a convenience sample of US parents reported that their child was more sad, depressed, or lonely since the onset of the pandemic.^3^ Longitudinal data from children in China aged 9 to 16 years showed an increase in depressive symptoms and suicidal thoughts and behaviors compared with before the implementation of COVID-19 home isolation restrictions.^4^ Potential pathways through which remote schooling could be negatively affecting child mental health include loss of social connection; decreased access to mental health services^5,6^; lack of identification, reporting, and support for youth experiencing abuse or maltreatment at home^7,8^; heightened psychosocial consequences of missed free and reduced price meals^9^; disrupted routines and lack of structure^10^; and stress related to online learning. Older children, who are forming more complex social relationships and navigating more high stakes academic outcomes, may be at heightened risk.

Youth may be uniquely susceptible to negative mental health outcomes if they are experiencing pandemic-related disruptions to in-person schooling in intersection with other adverse circumstances, such as racism, poverty, food insecurity, or home instability.^11^ Loss of access to school-based mental health care may be of heightened importance for youth from low-income families, as they are most likely to receive mental health services solely from their school.^12^ Difficulty accessing and engaging with distance learning due to technological limitations is most likely for youth from families with the lowest income,^13,14^ which may contribute to inequitable distress during remote learning. Most families are struggling to support their child’s at-home learning^15^; however, challenges may be heightened among families with low income who lack workplace flexibility or the financial resources to work fewer hours or pay for child supervision.^13^ One approach that 6% of families in the United States have taken to supporting their children and reducing parental demands related to remote instruction is forming what are known as pods with other families,^11^ where groups of children complete remote schooling activities together. The extent to which pods offset potential negative mental health consequences of remote schooling remains unclear.

Debates are ongoing about the costs and benefits of school closures during the COVID-19 pandemic.^1^ Simulation modeling raises concerns that school closures may catalyze negative child health and economic outcomes across the lifespan.^16,17^ Obtaining reliable data about the extent to which different instructional modalities are associated with key youth outcomes is critical for informing such models. This can help guide decision-making at a local and national level as the current pandemic progresses and during future pandemics. Youth mental health is a key outcome to consider.^18^ Most studies to date have used convenience samples that do not reflect the full US population. In a large and nationally representative sample of parents of school-aged youth in the United States, the present study sought to characterize the sociodemographic patterning of school modality, assess the extent to which school modality was associated with youth mental health outcomes, and describe how these associations varied by child age and socioeconomic status.

## Methods

### Study Design, Setting, and Population

This study is a nationally representative cross-sectional survey of US adults aged 18 to 64 years with at least 1 child aged 4 to 17 years in the household. The protocol was approved by the Seattle Children’s institutional review board. Potential participants reviewed a study information sheet (verbally or online, depending on data collection modality) before consenting to participate. A waiver of documented consent was obtained. The study follows the Strengthening the Reporting of Observational Studies in Epidemiology (STROBE) reporting guideline.

Participants were selected from NORC’s AmeriSpeak Panel. AmeriSpeak is a probability-based panel and provides coverage of approximately 97% of the US household population. Randomly selected US households were sampled using area probability and address-based sampling, with a known, nonzero probability of selection from the NORC National Sample Frame. While most AmeriSpeak households participate in surveys by web, households without internet can participate by telephone. Surveys were offered in English and Spanish by NORC personnel.

Panel participants were selected from a total of 36 sampling strata based on age, race and Hispanic ethnicity, education, and gender. The size of the selected sample per stratum was determined by the population distribution for each stratum. Sample selection accounted for expected differential survey completion rates by demographic groups so that the set of panel members with a completed interview was representative of the target population. One adult per household was eligible for selection. For questions about children, parents and guardians were asked to reference the child with the closest upcoming birthday.

Survey responses were fielded between December 2 and December 21, 2020. Panelists were offered the cash equivalent of $3 for completing the survey. In total, 9115 panelists were invited to participate; 2693 (29.5%) completed the screener; of them, 2530 (94.0%) were determined eligible; and 2324 eligible panelists (91.9%) completed the survey, 2299 via the internet and 25 via the telephone. Eleven respondents who did not provide information on schooling modality were excluded.

### Measures

#### Strengths and Difficulties Questionnaire

The parent-report version of the Strengths and Difficulties Questionnaire (SDQ) was used to assess child mental health difficulties in 4 domains: emotional problems, peer problems, conduct, and hyperactivity. Questions asked parents to reference their child’s behavior over the past month, with response options of 0, indicating not true; 1, indicating somewhat true; and 2, indicating certainly true. Age-specific versions were used depending on the age of the reference child. Responses from these 4 subscales were summed to provide a total difficulties score that ranged from 0 to 40, with higher scores indicating greater difficulties.^19^ Measurement reliability was good (McDonald ω = 0.85). We used Cohen’s guidelines for clinical significance,^20^ with small (*d* = 0.2), medium (*d* = 0.5), and large (*d* = 0.8) standardized mean differences corresponding to SDQ total score differences of 1.3, 3.3, and 5.2 points, respectively.

#### Schooling Modality

Parents were asked whether their child attended school fully remotely, fully in person, or in a hybrid format during past month. Parents whose children attended school remotely or in a hybrid format were also asked whether their child was part of a learning pod with other children during the school day; children attending school fully in person were assumed not to be in a pod.

#### Demographic Characteristics

Demographic characteristics included child age, gender, household income, parent race and ethnicity, and parent education. Income was measured in 18 categories ranging from less than $5,000 per year to $200,000 or more per year and analyzed as a continuous variable using the midpoint of each category. Participants could select from the following racial and ethnic groups: White, non-Hispanic; Black, non-Hispanic; other, Non-Hispanic; Hispanic; 2 or more races, non-Hispanic; and Asian or Pacific Islander, non-Hispanic. Race and ethnicity were analyzed in this study due to the concern that school closures were inequitably impacting racial and ethnic minority groups.

### Statistical Analysis

Patterning by sociodemographic characteristics was assessed using Wald tests within a generalized linear modeling framework, with appropriate link functions for categorical variables. Linear regression was used to estimate the covariate-adjusted association between schooling modality and child mental health outcomes. To assess how this association varied across child age and household income, interaction terms were computed and centered at target population means. Standardized effect sizes were calculated by standardizing the total difficulties score and the continuous factors (ie, income, child age), leaving binary indicators untransformed. Post hoc analyses were conducted to examine whether child gender or the presence of more than 1 child in the home moderated the effects of schooling modality. Exploratory analyses were conducted to examine whether schooling modality was more strongly associated with differences in subcomponents of the total difficulties score, using the Benjamini-Hochberg adjustment with the false discovery rate set to 5% to manage multiple testing.

The target population for this analysis was households with at least 1 child aged 4 to 17 years. Statistical weights were initially calculated using panel-based sampling weights, then raked to external population totals associated with age, sex, education, race and Hispanic ethnicity, housing tenure, telephone status, and Census division. External population totals were obtained from the Current Population Survey, March 2020. Weights were applied to all statistical models. All calculations were performed in R version 3.6 (R Project for Statistical Computing).^21^ Statistical significance was set at *P* < .05, and all tests were 2-tailed.

## Results

### Sample Characteristics

Participants were closely representative of all US households with children (eFigure 1 in the Supplement) in terms of race (Black participants: unweighted percentage, 10.5% [244 participants] vs benchmark percentage, 12.5%) and ethnicity (Hispanic participants: unweighted percentage, 16.0% [372 participants] vs benchmark percentage, 24.8%), with undersampling of parent education (≤high school equivalent education: unweighted percentage, 18.1% [421 participants] vs benchmark percentage, 38.6%) and oversampling of women (unweighted percentage, 71.9% [1671 participants] vs benchmark percentage, 53.7%) and participants with low income (annual household incomes <$30 000: unweighted percentage, 21.3% [495 participants] vs benchmark percentage, 9.9%). Children were nearly equally male (1119 [48.0%]) and female (1163 [50.1%]), and child age was dispersed evenly from preschool through high school age groups (mean [SD] age, 10 [4] years).

### Sociodemographic Patterning of School Attendance

Overall, 1340 children (58.0%) attended school remotely, whereas 415 (18.0%) attended school in a hybrid format, and 556 (24.1%) attended school fully in person. A total of 229 children attending remote school (17.1%) and 121 children attending school in a hybrid format (29.3%) belonged to a learning pod. Sociodemographic patterning (Table) was similar between the in-person and hybrid modalities, but fully remote schooling was strongly patterned along lines of parent race and ethnicity as well as income. Parents of 336 children attending school in person (65.8%) but of 597 children attending school fully remotely (44.5%) were White, whereas all other racial/ethnic groups had larger proportions of children attending school fully remotely (*P* < .001). Children attending school remotely came from households with approximately $10 000 less in yearly income than children in other schooling modalities (mean difference, −$9719; 95% CI, −$15 111 to −$4327; *P* < .001).

**Table.** Demographic Characteristics by Schooling Modality

Characteristic	Participants, weighted No. (%)			*P* value
	In-person school	Remote school	Hybrid	
Overall	556 (24.1)	1340 (58.0)	415 (18.0)	NA
Child age, mean (SD), y	9.1 (3.9)	10.4 (4.2)	10.8 (4.0)	<.001
Child gender				
Male	272 (48.8)	623 (46.6)	213.8 (51.5)	.33
Female	280 (50.4)	687 (51.3)	189.7 (45.7)	
Other	0	3 (0.2)	5 (1.1)	
Prefer not to answer	4 (0.8)	25 (1.8)	7 (1.6)	
Child belongs to a learning pod	NA	229 (17.1)	121 (29.3)	<.001
Parent race/ethnicity				
White	366 (65.8)	597 (44.5)	259 (62.3)	<.001
Hispanic	101 (18.2)	405 (30.2)	74 (17.9)	
Black	55 (9.9)	187 (13.9)	38 (9.2)	
Asian	16 (2.9)	82 (6.1)	21 (5.0)	
≥2, non-Hispanic	12 (2.2)	45 (3.4)	10 (2.5)	
Other, non-Hispanic^a^	6 (1.1)	25 (1.8)	13 (3.0)	
Parent education				
<High school	40 (7.2)	176 (13.1)	62 (14.8)	.14
High school graduate	161 (29.0)	379 (28.2)	84 (20.3)	
Vocational, technical school, some college, or associates’ degree	152 (27.3)	370 (27.6)	116 (27.9)	
Bachelor’s degree	118 (21.2)	250 (18.7)	90 (21.7)	
Postgraduate study or professional degree	85 (15.3)	166 (12.4)	63 (15.2)	
Household income, mean (SD), thousands of $	74.8 (5.5)	65.0 (5.3)	77.4 (5.8)	.003

Abbreviation: NA, not applicable.

^a^ Other was included as a survey option and concatenated with the Asian or Pacific Islander, non-Hispanic category.

### Schooling Modality and Child Mental Health Outcomes

**Figure 1.**
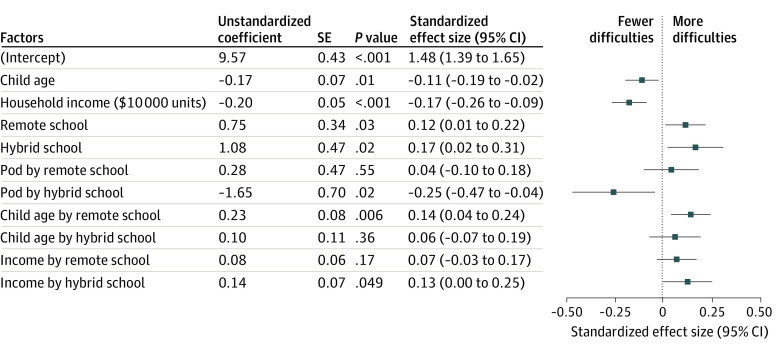
Adjusted Associations of Mental Health Difficulties With Schooling Modality and Child and Family Characteristics Regression models were weighted to reflect the target population. Regression models controlled for race and ethnicity and parent education (not shown). The main effect size of learning pod status was excluded because it was aliased, meaning it took the value of 0 for all children attending in-person schooling, so effect sizes are all interpreted as specific to each learning modality.

**Figure 2.**
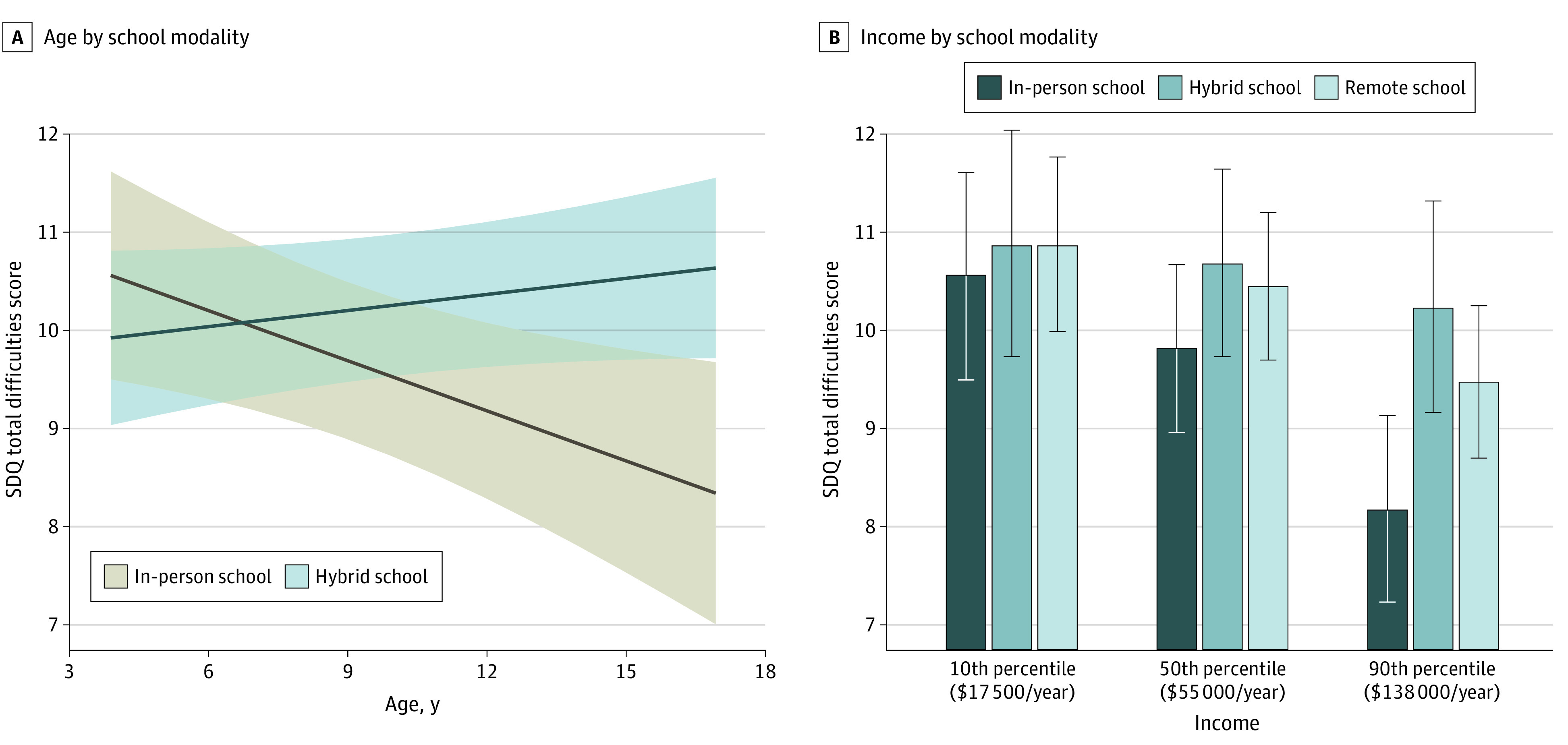
Estimated Total Difficulties Scores Across Age and Household Income Levels SDQ indicates Strengths and Difficulties Questionnaire.

**Figure 3.**
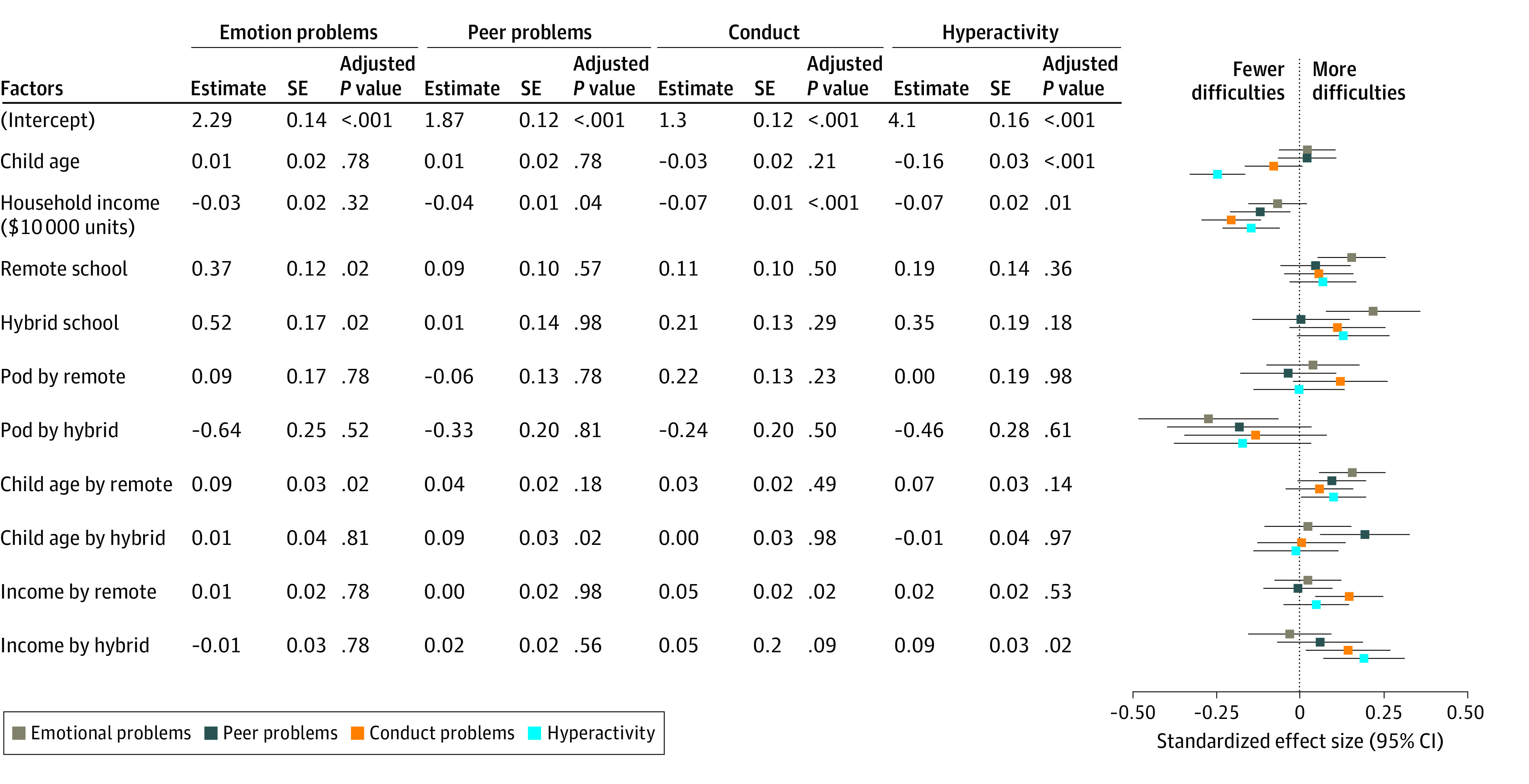
Adjusted Associations of Mental Health Difficulty Component Scores With Schooling Modality and Child and Family Characteristics *P* values are Benjamini-Hochberg–adjusted *P *value, with the false discovery rate set to 5%.

A significant interaction between schooling modality and age indicated that older children attending school remotely had more difficulties compared with those attending in-person (standardized effect size, 0.23 [95% CI, 0.07 to 0.39] per year of child age; *P* = .007) (Figure 1). This result implies that a child aged 17 years attending school remotely would be expected to have a total difficulty score 2.4 points higher than a child of the same age attending school in person, corresponding to a small effect size in favor of in-person schooling (Cohen *d* = 0.35 [95% CI, 0.14 to 0.56]) (Figure 2A; eFigure 2 in the Supplement). Conversely, a child aged 4 years attending school remotely would be expected to have a total difficulty score 0.5 points lower than a child of the same age attending school in person, corresponding to a very small effect size in favor of remote schooling (*d* = −0.10 [95% CI, −0.27 to 0.07]). The association of age with mental health outcomes in hybrid schooling was not significantly different from either the in-person or remote modalities. Exploratory models (Figure 3) suggested that differences across schooling modalities were partially driven by older children in remote learning experiencing more emotion problems, although these effect sizes were small (estimated effect size [SE], 0.09 [0.03]; *P* = .02).

A significant interaction was also found between schooling modality and income level. Children from families with higher income benefitted more from attending schools in person compared with their peers from families with lower income (B = −0.20 [95% CI, −0.10 to −0.30] per $10 000-increase in annual income; *P* < .001). This advantage was not apparent for children attending hybrid school (B = −0.05 [95% CI, −0.16 to 0.06] per $10 000-increase in annual income; *P* = .34), and it was directionally lower but not significantly different for children attending remote school (B = −0.12 [95% CI, −0.04 to −0.20] per $10 000-increase in annual income; *P* < .001). Because income distributions were skewed, differences across schooling modalities were trivial for many children in the lower- and middle-income brackets, whose incomes were separated by smaller dollar amounts compared with those in the higher-income brackets (Figure 2B). For incomes at the 90th percentile ($138 000 per year), a child aged 10 years attending school in person was estimated to have a lower difficulty score than a child attending in the hybrid or remote modalities (remote schooling: *d* = 0.21; 95% CI, 0.06 to 0.37; hybrid schooling: *d* = 0.26, 95% CI, 0.07 to 0.45). Exploratory models suggested these differences were driven by decreases in hyperactivity (estimated effect size[SE], 0.09 [0.03]; *P* = .02) and conduct (estimated effect size [SE], 0.05 [0.02]; *P* = .02) for the hybrid and remote groups, respectively (Figure 3).

Learning pods fully buffered the associations of hybrid schooling (*d* = −0.25; 95% CI, −0.47 to −0.04) but not remote schooling (*d* = 0.04; 95% CI, −0.10 to 0.18) with negative mental health outcomes. In an exploratory post hoc analysis, neither child gender nor number of children in the household moderated the association between schooling modality and total difficulties.

## Discussion

Decisions about school closures in the context of COVID-19 and during potential future pandemics need to balance risks and benefits.^22^ The consequences on child mental health are a potential risk, and data on the mental health outcomes of remote schooling can inform this decisional calculus. To that end, we examined differences in the sociodemographic patterning of school closures and child mental health outcomes using a large, nationally representative sample of households with children aged 4 to 17 years. The association between remote schooling and child mental health varied by child age and, to a lesser extent, household income.

Older children who attended school remotely had worse mental health outcomes compared with those who attended school in person, whereas younger children who attended school remotely had comparable or slightly better mental health outcomes than those who attended in person. Understanding who is most at risk for poor mental health may help to inform resource allocation during and after the COVID-19 pandemic. Thus far, reopenings have prioritized younger children because of the lower transmission rates compared with older students^23^ and the relatively higher burden of care they require from working parents. Unfortunately, current results suggest that school closings have been disproportionately detrimental to mental health adjustment for older children, with effect sizes in the small range.

Both now and as children fully return to school, older children attending school remotely may be most in need of targeted support. Although one logical source of support is community and school-based mental health resources, further information on the pathways driving mental health differences is critical for informing approaches to intervention. For example, our analysis suggested that emotional difficulties accounted for the largest proportion of mental health challenges. Emotional problems due to decreased social connection or disrupted health behavior routines, such as decreased physical activity, may be remedied naturally as school returns to in-person learning. Worries stemming from valid concerns about learning loss during remote schooling may be better served by academic than mental health interventions. Of note, learning pods buffered the negative associations of hybrid schooling but not remote schooling. Although the reason for this cannot be conclusively determined from the present data, one possibility is that learning pods build most effectively on educational engagement developed during in-person instruction, keeping children engaged and supporting them to transition effectively across the hybrid modalities.

The mental health sequelae associated with school closures were patterned along sociodemographic lines in 2 ways. First, children from lower-income families, along with children of Black and Hispanic parents, were much less likely to attend school in person than their peers from higher-income families or with White parents, consistent with prior research.^2^ Because school closures were generally associated with worse outcomes—in particular for older children—this elevated likelihood of remote learning suggests that school closures were also associated with racial and ethnic disparities in mental health outcomes. Such differences in school modality may in part stem from differences in available options. Prior research has found that private schools, which are disproportionately populated by non-Hispanic White children from relatively affluent families, were more likely to open during the pandemic than public schools.^2^ Differences may also stem from structurally determined demographic patterning in family decision-making. Prior findings suggest that less affluent and Black and Hispanic families have lower confidence that their child’s school can adequately mitigate COVID-19 risk.^24^ They are also more likely to live in a household with a high-risk family member.^24,25^ As a result, they may be more likely to choose remote instruction when given a choice of modalities.

The second way in which mental health sequelae associated with school closures were patterned along sociodemographic lines was the strength of the benefits of in-person instruction. Higher family income was associated with greater benefit from attending schools in person compared with lower income, with exploratory analyses suggesting differences were driven by conduct and hyperactivity. It is possible that schools with more resources have smaller class sizes, closer student-teacher relationships, more school counselors per student, and consequently, a greater ability to help regulate child behavior.^26^ School funding decisions that allow schools to safely reopen can help minimize disparities in child mental health.

Decreasing pediatric mental health inequities is a problem that predates the COVID-19 pandemic. Accessing community-based services during school closures may be challenging for financial reasons or due to a lack of preexisting relationships with caregivers.^5^ Thus, fostering the use of existing school-based resources is critical. However, during the COVID-19 pandemic, school counselors have spent less time working directly with students in part because they have been asked to fill logistical or administrative roles during rapid school pivots to remote instruction.^27^ Adequate funding and role prioritization is necessary for trained clinical personnel in schools to be able to focus on providing mental health care for students. Critically, staffing for counselors, social workers, and other clinical personnel was typically inadequate even before the pandemic.^28^ Responding to the consequences of the COVID-19 pandemic presents an opportunity to make longer-term changes that strengthen school funding for mental health personnel. Beyond personnel, there may be other organizational opportunities to strengthen the connection to school-based and community-based mental health supports during remote learning and once students return to the classroom. This could include building routinized pathways to connect students to school counselors (eg, through screening or routine check-ins) and testing novel and low-resource forms of mental health support, such as single-session online interventions.^29^

### Limitations

This study has limitations. It was an observational study, limiting our ability to make causal interpretations. In particular, the survey did not differentiate whether remote schooling was optional or mandatory, raising the possibility of children selecting their schooling modality based on other characteristics that could be associated with mental health difficulties, such as having a family member with high medical risk or distrusting the school’s ability to keep them safe.^24^ We also omitted other potentially important explanatory variables, either because we did not collect information on them or were underpowered to analyze them. These include child race and ethnicity, the number of caregivers in the home, whether children attended private school, and other metrics to gauge school resources. Future research examining the mechanisms that transmit the association between remote schooling and mental health difficulties (eg, decreased socialization vs learning loss) will help to inform intervention strategies.

## Conclusions

This study found that attending school remotely during the COVID-19 pandemic was associated with disproportionate mental health consequences for older and Black and Hispanic children as well as children from families with lower income. In the context of complex school reopening decisions that balance competing risks and benefits, these findings suggest that allocating funding to support safe in-person instruction may reduce mental health inequities associated with race/ethnicity and income. Critically, as children return to in-person instruction, mental health inequities may not resolve on their own. Ensuring that all students have access to additional educational and mental health resources must be an important public health priority, met with appropriate funding and work force augmentation, during and beyond the COVID-19 pandemic.

## Notes

Supplement. **eFigure 1.** NORC Final Report **eFigure 2.** Estimated Total Difficulties Scores Across Age by Schooling Type
