# Errors in Table and Figure

**DOI:** 10.1001/jamanetworkopen.2021.32935

**Published:** 2021-09-30

In the Original Investigation “The Association Between School Closures and Child Mental Health During COVID-19,”^1^ published September 3, 2021, there were errors in Table 1 and Figure 2. In Table 1, the SDs for household income should have been 54.6 for participants attending in-person school, 53.4 for participants attending remote school, and 58.1 for participants attending hybrid school. In Figure 2A, the labels should have read “In-person school” and “Remote school.” This article has been corrected.^1^
