# Supplementary material for: The Association Between School Closures and Child Mental Health During COVID-19
Source: JAMA Netw Open. 2021 Sep 3;4(9):e2124092. doi: 10.1001/jamanetworkopen.2021.24092 (PMC8417763; doi:10.1001/jamanetworkopen.2021.24092)
Supplement: Supplement. — eFigure 1. NORC Final Report eFigure 2. Estimated Total Difficulties Scores Across Age by Schooling Type [file jamanetwopen-e2124092-s001.pdf]

Supplemental Online Content

Hawrilenko M, Kroshus E, Tandon P, Christakis D. The association between school closures and child mental health during COVID-19. *JAMA Netw Open.* 2021;4(9):e2124092. doi:10.1001/jamanetworkopen.2021.24092

eFigure 1. NORC Final Report

eFigure 2. Estimated Total Difficulties Scores Across Age by Schooling Type

This supplemental material has been provided by the authors to give readers additional information about their work.

© 2021 Hawrilenko M et al. *JAMA Network Open.*

eFigure 1. NORC Final Report

AmeriSpeak | AAPOR Transparency Initiative | NORC CARD | Final Report

COVID-19 Parents Study

January 28, 2021

Panel Outcomes

Weighted HH Recruitment Rate | Weighted HH Retention Rate

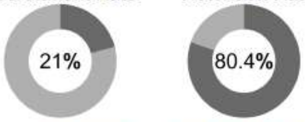

Survey Overview

Study Population: US household with children age 4-17
Sample Units: 9115
Completed Units: 2324
Expected Eligibility Rate: 85%
Observed Eligibility Rate: 94%
Margin of Error: ±2.91 percentage points (pp)
Avg. Design Effect: 2.05
Survey Field Period: December 2, 2020 - December 21, 2020
Median Duration (minutes): 15
Prepared for: Seattle Children's Hospital

Survey Outcomes

Screener Completion Rate | Survey Completion Rate | Weighted Cumulative Response Rate

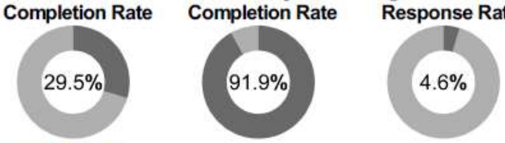

Benchmark Comparisons

	Unweighted (%)	Weighted (%)	Benchmark (%)	Difference [2] (pp)
**Household Income [1]**				
Less than $29,999	21.3	26.8	9.9	16.9
$30,000 to $74,999	36.5	36.7	27.7	9.0
$75,000 to $124,999	26.5	23.4	25.6	-2.2
$125,000 Plus	15.7	13.1	36.8	-23.7
**Member Age**				
18 - 34	23.5	33.1	33.3	-0.2
35 - 44	41.3	49.9	49.9	0.0
45 - 64	35.2	17.0	16.8	0.2
**Member Race/Ethnicity**				
White	63.9	52.8	52.7	0.1
Black	10.5	12.1	12.5	-0.4
Hispanic	16	25.0	24.8	0.2
Asian/Pacifi	3.8	5.1	7.5	-2.4
Others	5.7	4.9	2.5	2.4
**Member Education Status**				
Less than High School	4.8	11.9	12.1	-0.2
High School Equivalent	13.3	27.2	26.5	0.7
Some College/Associate De	38	27.6	27.7	-0.1
Bachelor's Degree	25.7	19.8	20.6	-0.8
Graduate Degree	18.2	13.5	13.2	0.3
**Household Ownership**				
Owner Occupied	63.2	58.8	70.0	-11.2
Renter Occupied/Other	36.8	41.2	30.0	11.2
**Children in Household**				
With 1+ Under 18 Years	100	100.0	100.0	0.0
**Household Marital Status**				
Currently Married	68	64.3	63.6	0.7
Separated/Divorced/Widowe	32	35.7	36.4	-0.7
**Sex**				
Male	28.1	46.5	46.3	0.2
Female	71.9	53.5	53.7	-0.2

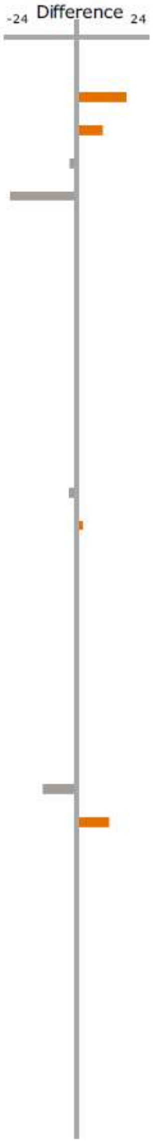

[1]Race/Ethnicity, Household Ownership, Income, Education, Number of Children, Marital Status, Gender, and Geographic Region benchmarks are from the March 2020 Census Bureau Current Population Survey. [2] The difference between the Weighted and Benchmark columns.

© 2021 Hawrilenko M et al. *JAMA Network Open.*

Glossary

Overview Section

Study Population: The total set of individuals of interest to which the researcher intends to apply their conclusions.

Sample Units: The number of panel members selected into the study sample.

Completed Units: The number of sample units that completed the interview based on the study-specific definition of what constitutes a complete interview.

Expected Eligibility Rate: The percentage of the sampling population who are expected to meet study eligibility criteria.

Observed Eligibility Rate: The percentage of the sample members who were eligible for the study among those who answered the screening questions.

Margin of Error: Margin of error is defined as half the width of the 95% confidence interval for a proportion estimate of 50% adjusted for design effect. It is therefore the largest margin of error possible for all estimated percentages based on the study sample.

Design Effect The design effect is the variance under the complex design divided by the variance under a SRS (simple random sampling) design of the same sample size. Design effect is variable-specific and the reported value is the median design effect calculated for a set of key survey variables.

Survey Field Length: the period from the earliest to the latest contact dates of cases sampled for the survey.

Duration: Length of time for completed interviews. Interview length is calculated differently depending upon whether the interview was conducted over the phone or via web. For telephone mode, it is the time from when the respondent picks up the telephone until they hang up the telephone. For web interviews, it is the time from when they first connect to the web system to the time they log off the system or become inactive. In the case of multiple contacts, this numberrepresents the sum of those contacts.

Benchmark Comparison Section

We compare demographics (from the CPS) to those of our survey respondents, both on a weighted and unweighted basis, to show how closely AmeriSpeak respondents represent the demographics of the study population overall.

We also compare study specific benchmarks (not available on all surveys) to those of our survey respondents to show how closely survey responses to key questions match to benchmarks from external surveys.

Panel Outcomes

Weighted Household (HH) Recruitment Rate: The weighted AAPOR RR III for the AmeriSpeak panel recruitment corresponding to the recruitment cohorts sampled for the study. A recruited household is a household where atleast one adult successfully completed the recruitment survey and joined the panel.

Weighted Household (HH) Retention Rate: The weighted percent of recruited households that are still available for sampling for this survey among the recruitment cohorts sampled for the study.

Survey Outcomes

Screener Completion Rate: The percent of sampled memberswho completed the screening questions and therefore with known eligibility status for the study.

Survey Completion Rate:

- The percent of sample members who completed thesurvey interview (for studies without screener)
- The percent of eligible sample members who completed the survey interview (for studies with screener).
- For a follow-up study: it is the percent of follow-uprespondents among baseline respondents.

Weighted Cumulative Response Rate: The overall survey response rate that accounts for survey outcomes in all response stages including panel recruitment rate, panel retention rate, and survey completion rate. It is weighted to account for the sample design and differential inclusion probabilities of sample members.

© 2021 Hawrilenko M et al. *JAMA Network Open.*

eFigure 2. Estimated Total Difficulties Scores Across Age by Schooling Type

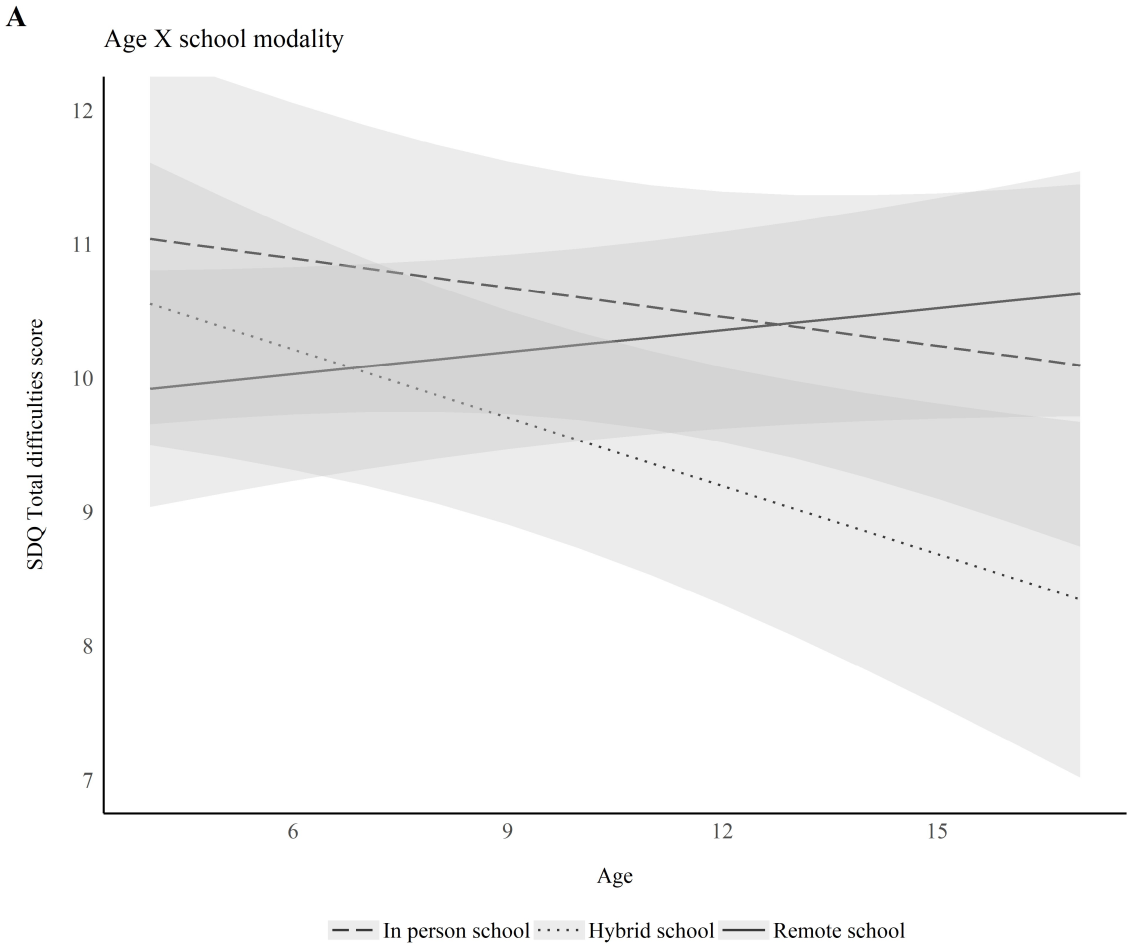

Note. Estimated values adjust for all model variables. The shaded region represents 95% confidence intervals.

© 2021 Hawrilenko M et al. *JAMA Network Open.*
